# Conceptualizing and Measuring Appetite Self-Regulation Phenotypes and Trajectories in Childhood: A Review of Person-Centered Strategies

**DOI:** 10.3389/fnut.2021.799035

**Published:** 2021-12-22

**Authors:** Alan Russell, Rebecca M. Leech, Catherine G. Russell

**Affiliations:** ^1^College of Education, Psychology and Social Work, Flinders University, Bedford Park, SA, Australia; ^2^School of Exercise and Nutrition Sciences, Institute for Physical Activity and Nutrition (IPAN), Deakin University, Geelong, VIC, Australia

**Keywords:** phenotypes, appetite regulation, mixture models, developmental trajectories, latent class analysis, unobserved (or underlying) heterogeneity, eating behavior, child

## Abstract

This review uses person-centered research and data analysis strategies to discuss the conceptualization and measurement of appetite self-regulation (ASR) phenotypes and trajectories in childhood (from infancy to about ages 6 or 7 years). Research that is person-centered provides strategies that increase the possibilities for investigating ASR phenotypes. We first examine the utility of examining underlying phenotypes using latent profile/class analysis drawing on cross-sectional data. The use of trajectory analysis to investigate developmental change is then discussed, with attention to phenotypes using trajectories of individual behaviors as well as phenotypes based on multi-trajectory modeling. Data analysis strategies and measurement approaches from recent examples of these person-centered approaches to the conceptualization and investigation of appetite self-regulation and its development in childhood are examined. Where relevant, examples from older children as well as developmental, clinical and educational psychology are drawn on to discuss when and how person-centered approaches can be used. We argue that there is scope to incorporate recent advances in biological and psychoneurological knowledge about appetite self-regulation as well as fundamental processes in the development of general self-regulation to enhance the examination of phenotypes and their trajectories across childhood (and beyond). The discussion and conclusion suggest directions for future research and highlight the potential of person-centered approaches to progress knowledge about the development of appetite self-regulation in childhood.

## Introduction

In current food environments, where there is an abundance of palatable but unhealthy foods, it is important that children are able to self-regulate appetite. Appetite self-regulation (ASR) means that children are better able to resist tempting but unhealthy foods, better balance energy intakes with expenditure and select and consume healthier diets. The conceptualization and measurement of children's ASR is an emerging field and new approaches are needed. The recent application of person-centered approaches to research on children's eating and ASR provide new perspectives and insights and is the focus of the present review.

There has been some vagueness and uncertainty about definitions of ASR in childhood, including its components and fundamental processes. A number of theoretical models together with potentially relevant constructs are evident in the literature. There is a general acceptance that ASR has to do with responding to hunger cues as well as to cues of satiety and satiation (1, 2). Satiation and satiety have been conceived as separate but overlapping processes. Satiation leads to the termination of eating while satiety is a post-consumption process that leads to the inhibition of further eating and is an ingredient in the inter-meal interval (3–6). Beyond that, it has been variously treated as a multidimensional construct that includes traits (e.g., food responsiveness or satiety responsiveness), processes [e.g., as in the Satiety Cascade (4, 7)] and individual skills or strategies (e.g., delay of gratification) (8). Many of the traits have been associated with the subscales of the Children's Eating Behavior Questionnaire (CEBQ). This scale includes food approach behaviors such as food responsiveness (e.g., child is attracted to food and eating) and food avoidance behaviors such as satiety responsiveness (e.g., child gets full easily and leaves food on his/her plate) (9). The Satiety Cascade is a model that involves pre-consumption processes (such as hunger cues and food responsiveness), processes during consumptions such as satiety and habituation, and post-consumptions processes such as satiety cues.

The conceptualization of ASR has been assisted by the application of overall models such as the bottom-up, top-down model (10–12) or dual processing model (13) and the satiety cascade (4, 7). These models incorporate aspects of traits, processes, and skills. In the bottom-up, top-down model there is a recursive interplay between bottom-up reactive, often emotion driven and automatic, approach or avoidance behaviors (such as hedonic responses to hunger or food cues) and top-down regulatory control processes including inhibitory control. In the dual processing model, self-regulation is conceived as involving an interaction between regulatory processes such as inhibitory control (top-down) and approach-avoidance behaviors (bottom-up). Here, self-regulation is considered to involve an interplay between impulse generating and impulse controlling systems. It is possible to group many of the ASR-related constructs that have been described and investigated in the literature under the main headings of bottom-up (approach), such as food responsiveness, bottom-up (avoidance) such as food fussiness, and top down such as inhibitory control (12). The bottom-up, top-down model for conceptualizing ASR in childhood is helpful in the investigation of ASR phenotypes as it provides a framework for the interpretation of patterns of characteristics and behaviors.

Most of the existing research on the development of ASR has taken a variable-centered approach [c.f. the studies reviewed in (8)]. Similarly, the conceptualization of fundamental processes in ASR such as in the models described above has been substantially based on variable-centered research. To broaden knowledge about ASR in childhood, there is a need to give greater recognition to the nature and extent of individual differences in behaviors, processes, traits, skills and trajectories in the development of ASR. There is limited research and theory about ASR that focuses separately on traits, processes, and skills. Instead, consistent with the multidimensional treatment of ASR in infancy and childhood, research designs typically include measures of one or more of traits, processes, and skills although with a heavy emphasis on traits. In the present review, the focus is on possible advances in conceptualization that could be gained from a person-centered perspective to research and theory. We discuss ASR phenotypes, trajectories of individual ASR indicators, and trajectories of underlying ASR phenotypes. We argue that the exploration of ASR phenotypes and trajectories has implications for the conceptualization and measurement of ASR in childhood. If it can be assumed that scholarship is at a relatively early stage of developing an integrated model of ASR in childhood, person-centered strategies could provide insights into how traits, processes and skills might be organized and interrelated.

The initial scoping of the review was based on keyword and abstract literature searches in the main databases: PubMed, PsycINFO, SCOPUS, Web of Science, and Google Scholar. The searches covered theoretical articles (e.g., person-centered vs. variable centered), data analysis (e.g., latent class analysis, trajectory analysis), and research evidence (e.g., eating behavior phenotypes, latent class, and profile analyses of eating behaviors in childhood). We sourced articles under these headings from the literature on children's eating and ASR, as well as other areas of scholarship (such as clinical psychology, developmental psychology, and educational psychology). In contrast to the structured approach of a systematic review consistent with our purpose we chose articles for their illustrative significance in relation to the purpose of discussing the nature, role, and contribution of person-centered strategies in the investigation of ASR phenotypes and trajectories in childhood.

We begin with a discussion of person-centered vs. variable-centered strategies before considering the prospect for investigations of phenotypes to examine ASR components and processes in childhood. The main body of the review is then about the measurement of trajectories and the potential of this approach to contribute to knowledge about the development of ASR.

### Person-Centered vs. Variable-Centered Approaches

Person-centered and variable-centered approaches answer different research questions, and can provide complementary information about a research field, including about child development (14–21). Variable-centered approaches (e.g., regression analysis, factor analysis, structural equation modeling) examine associations between variables in a population and are suited to questions about normative development and the effect of one variable on another, especially in terms of the contributions of predictor variables to outcome variables (16, 22–26). In this case, data are aggregated and individual differences are treated as “noise” or “errors” which provides more parsimony (19) but less specificity (16).

In contrast, individual differences, or an assumption that the sample is not uniform and that behavior and psychological processes are unique to the individual or differ from one group to another (15, 27), are the basis of person-centered approaches such as cluster analysis and finite mixture models (14, 15, 20, 27–31). In this case the emphasis is on individual and sub-group differences within the sample, including sub-group differences in development. Person-centered approaches are helpful for understanding possible developmental mechanisms (20) and allow investigation of inter-individual (between-person) differences in intraindividual (within-person) profiles of behavior or change (32, 33). Person-centered approaches categorize participants into subgroups, or phenotypes, based on a set of shared characteristics or attributes. The resulting phenotypes may be based on complex patterns of substantive variables to provide a more detailed and nuanced picture of development. For these reasons, person-centered approaches have the potential to advance knowledge about ASR and its development in childhood.

### The Contribution of Phenotypes to the Conceptualization of ASR

Technically, a phenotype is a set of behaviors and characteristics arising from the interaction of the genotype with the environment (34). The phenotype concept is useful as a way of describing combinations of individual traits and behavior, especially as they apply to sub-groups of the population. The concept has been applied to children's eating behaviors as we discuss here, but also to parent feeding practices (35, 36), in developmental psychology (37–39), and in other areas of psychology such as educational psychology (40) and clinical psychology (41–43).

The examination of ASR phenotypes can contribute to the understanding of components and processes in ASR. The investigation of possible phenotypes enables ASR to be conceptualized in new ways by suggesting different combinations of measures of traits, processes, skills, behaviors and other ASR-related measures (e.g., neurobehavioral indicators) and how these combinations interact for separate sub-groups. For example, ASR difficulties could be associated with increased food approach tendencies in some groups of children, difficulties in responding to satiety and satiation cues in other groups, and increased impulsiveness and reduced inhibitory control in other children, or different combinations and patterns of change of these characteristics. The so-called patterns of change could vary from consistency across ages, to increases, decreases or other variations such as consistency followed by an increase or a decrease. Possible insights from the examination of phenotypes have been illustrated in an adult sample where four phenotypes of obesity-related behaviors and characteristics were derived from behavioral and questionnaire measures (44). One phenotype, labeled “hungry brain,” was described as having abnormal satiation, and another phenotype, labeled “emotional eating,” was high on hedonic eating. The other phenotypes were “hungry gut,” involving abnormal satiety, and “slow burn” described as involving decreased metabolic rate. The authors assumed that the phenotypes revealed something about fundamental processes in weight gain and obesity and described them as “actionable” because they were helpful to target weight-loss treatments. The examination of phenotypes has been enhanced by the application of person-centered data analysis strategies. In the following section we describe the analytical strategies available for examining phenotypes and illustrate their use in ASR/eating behavior research. These include strategies for analyzing both cross-sectional and longitudinal data.

## Person-Centered Approaches and Their Application in ASR Phenotype Research

Earlier person-centered approaches for evaluating the underlying psychological attributes of weight gain in cross-sectional studies included cluster analyses using hierarchical (e.g., Ward's) or partitioning (e.g., k-means) methods (45, 46). However, finite mixture models (FMM), also known as model-based clustering or latent variable mixture models (LVMM), are newer techniques that have become a popular alternative for understanding population heterogeneity (47). Examples of such techniques include latent class analysis (LCA), latent transition analysis (LTA), and growth mixture models (GMM) (47). FMM draw on a structural equation modeling (SEM) approach and encompass a “collection of statistical approaches” for analyzing cross-sectional and longitudinal data [(41, 48), p. 175]. They take one or more observed input variables to model the probability of participants belonging to latent (i.e., unobserved or underlying) subgroups and classify participants to the subgroup with the highest probability of their belonging (41, 48). The resultant subgroups may be referred to as classes, profiles, typologies, or phenotypes, with the latter more commonly used in the field of psychology (32). Interested readers are referred to Berlin and colleagues (18, 48) for an introduction and non-technical account of cross-sectional and longitudinal FMM/LVMM approaches, covering assumptions and a “how to” description of their use.

### Cross Sectional Studies: Latent Class and Latent Profile Analysis

LCA for categorical data and latent profile analysis (LPA) for continuous data are foundation person-centered strategies used in many fields including medical, biological, physical nutritional and social sciences (18, 31, 40, 48, 49). LCA and LPA are examples of FMM/LVMM used in cross-sectional studies (31) and identify latent subgroups based on specific combinations of observed variables (18, 48). The goal of the analysis is to determine the optimal number of latent subgroups that summarize the unique, and often complex, patterns of the observed variables within individuals (32).

In cross-sectional studies, distinct eating behavior subgroups, or phenotypes, have been identified in children using LCA or LPA. For example, Boutelle et al. (50) conducted LPA from multiple measures of eating behaviors in a sample of 8 to 12 year-old children with overweight or obesity. Three latent profiles were identified, labeled as (i) high satiety responsiveness, (ii) high food responsiveness and (iii) moderate satiety and food responsiveness. Although each phenotype was associated with overeating and overweight, the phenotypes involved combinations of different levels on the individual variables, such as eating in the absence of hunger (EAH), satiety responsiveness, food responsiveness, negative affect eating, loss of control eating and external eating.

In a sample of 4-year-old children, Tharner et al. (51) conducted LPA on Children's Eating Behavior Questionnaire (CEBQ) scores and identified six eating behavior profiles. Most children were in the “moderate eaters” (44.6%) or “avoidant eaters” (33.2%) profiles. The authors were mainly interested in the “fussy eater” profile (5.6%) which was characterized by high scores on the subscales of satiety responsiveness, food fussiness and slowness in eating combined with low scores on the enjoyment of eating subscale. This subscale was associated with dietary, weight and parental factors, demonstrating the utility of examining a number of eating behavior subscales as profiles rather than individual variables.

### Longitudinal Studies of Subgroups: Latent Transition Analysis

LTA is a longitudinal extension of LCA and LPA that identifies unique latent subgroups based on combinations of observed variables, and an individual's transition, or movement, between these latent subgroups over time (40, 52–55). LTA has mostly been applied to two time points, but three may be used (16, 56, 57). In longitudinal research, LTA may be used to investigate different developmental paths or transitions from one phenotype to another over time (16, 47, 53). It also enables the examination of whether phenotypes might be age-specific or if they are established early and then are maintained across childhood. Further, it could assist in the examination of outcomes or changes associated with interventions (e.g., whether participants transition to a different phenotype following intervention).

Pitt et al. (58) illustrate the use of LTA to examine developmental change. The authors calculated phenotypes (using LCA) for dietary patterns at age 3 and 5 years and then used LTA to investigate changes in the subgroups over time. Similarly, development change was explored by Swanson et al. (59) who measured eating disorder symptoms in girls in five age groups from preadolescence, early adolescence, late adolescence and two young adulthood periods and calculated transition probabilities from the latent classes at age 9–12 to classes at age 19–22. Latent transition probabilities following LCA has also been used to investigate learning outcomes (55) in a way that could provide a parallel for the examination of eating-based intervention outcomes. These approaches examine transitions between groups, however changes over time are also usefully explored with modeling of trajectories.

### Trajectories Modeling

Nagin (60) argued that charting and understanding trajectories in longitudinal research is fundamental to knowledge about development, including the possibility that sub-groups might follow distinct trajectories. Trajectory analyses in longitudinal research might inform the investigation of multifinality (a common starting point, but then divergence of trajectories), equifinality (different starting points, but convergence on a common end point, possibly via different routes or paths), and fanning (increasing interindividual differences in trajectories over time) (17, 61).

There are two kinds of analytical strategies for examining trajectories of ASR-related eating behaviors and the associated outcomes of weight gain, adiposity or BMI. The first involves the investigation of trajectories for individual variables. The second approach has been to examine trajectories that include multiple variables in the one analysis.

#### Longitudinal Studies of Trajectories of Single Variables: Latent Growth Mixture Modeling

GMM is a statistical approach for modeling the average rate of individual change, or trajectories, across three or more time points. Whilst LTA involves the identification of latent subgroups and then the calculation of transitions, GMM models include the trajectories when calculating the latent subgroups (16). GMM is an extension of the traditional latent growth curve model (62) and includes latent class growth analysis (LCGA), a simplified GMM, and group-based trajectory modeling (GBTM), a special case of LCGA that assumes error variances are the same for all latent subgroups (18, 21, 32, 33, 60, 63–68). Unlike conventional latent growth curve modeling which assumes the same pattern of growth corresponds to the whole population, GMM takes into account population heterogeneity and identifies latent subgroups of individual growth patterns, or developmental trajectories (62).

A number of authors compare the assumptions and use of the main approaches to GMM (18, 32, 43, 69). Nagin and Odgers (43) argue that while there are technical differences between these approaches (i.e., they make different assumptions about the distribution of trajectories in the population), they are all designed to assign individuals into trajectory groups. For example, LCGA is a restricted version of GMM that constrains the variations (i.e., variances and covariances) within each class to zero and as a consequence reduces the number of parameters and simplifies model selection (70). LCGA assumes that all individual growth trajectories within classes are homogeneous (71) and is often recommended as a first step in the exploration of possible latent classes. GMM relaxes the assumption that all individuals in a class are from a single homogenous population (67) and estimates all growth factors (e.g., means, variances and covariances). However, such increases in model complexity may lead to estimation difficulties, including non-convergence or non-optimal latent subgroup solutions; the model chosen should be one that best fits the data and leads to meaningful subgroup solutions based on substantive prior theory (62). Berlin et al. discuss when and how a researcher might use LCGA and GMM, including a step-by-step account of processes in the identification of latent trajectory subgroups (18, 48). After conducting Monte Carlo simulations of synthetic data, Den Teuling et al. (69) concluded that GMM provided the “best overall performance.”

A common example of phenotypes defined in terms of trajectories of ASR-related constructs with a single variable is trajectories for weight gain or BMI. Norris et al. (72), for instance, examined weight trajectories from 0 to 60 months of age using GMM to identify five groups of individuals with different average trajectories. The subgroups included “average,” “high-decreasing,” and “stable-high” BMI trajectories. They then examined maternal (e.g., maternal BMI and education), family and birth characteristics of children associated with the different trajectories. Becnal and Williams (64) and Ventura et al. (73) followed a similar approach, using GMM to identify several weight trajectories. Risk factors were included as covariates and health outcomes associated with the trajectories were examined. In a birth cohort, van Rossem et al. (74) used LCGA to investigate trajectories for BMI until age 11 years. Trajectories of persistent overweight and overweight reduction were subsequently found to be related to early-life and parent factors including parent overweight.

Trajectories of individual ASR-related eating behaviors have also been explored. Derks et al. (75) used LCGA to examine trajectories for individual CEBQ scales assessed at ages 4 and 10 years. They found three patterns for emotional overeating and five patterns for food responsiveness, but no subgroups for enjoyment of food and satiety responsiveness. Follow-up regression analyses enabled them to explore early life predictors of each of the trajectories. Herle et al. (70) also used LCGA to investigate trajectories of child eating behaviors in the first 10 years of life from parent reports. They reported a number of trajectories for each of the single variables of overeating, undereating and fussy eating. The eating trajectories were associated with later zBMI in meaningful ways and were also found to be predictive of later eating disorder diagnosis.

These examples illustrate the potential contribution of person-centered trajectory analyses to the identification of developmental patterns for weight gain and ASR-related eating behaviors. They also show how predictors or risk factors can be related to trajectories as well as relationships between trajectories and outcomes.

#### Longitudinal Studies of Multiple Trajectories: Multi-Trajectory Modeling

Rather than using a single trajectory variable to assign individuals to a phenotype, multi-trajectory modeling, or multivariate GBTM, approach defines trajectory phenotypes using multiple and distinct trajectories variables (76). It is a variation of univariate GBTM. It defines a trajectory group in terms of trajectories for multiple indicators and takes account of the interrelationships among the indicators in a multivariate design. Nagin et al. (76) provide two illustrative examples and argue there is a need to sharpen guidelines for model selection and evaluation. Their first illustrative example was from male subjects in the Dunedin Multidisciplinary Health and Development Study with measures at different ages from 3 to 38 years. There were five trajectory groups, defined by the patterns of trajectories on three physiological outcome variables. The second illustrative example was from the Montreal-based longitudinal study of 1,037 males with measures from ages 6 to 17 years. The analysis yielded five trajectory groups based on the pattern of trajectories of four individual variables.

We illustrate this multivariate approach to the investigation of latent trajectory phenotypes for ASR-related eating behaviors with two studies. First, Epstein et al. (77) measured trajectories of food habituation to salty, sweet and savory foods in a sample of 8–12 year-old children. GBTM was used to identify individual trajectory phenotypes for the three foods and multivariate GBTM was used to determine trajectories for the combination of foods. The habituation phenotypes (such as “rapidly decelerating habituation” vs. “slower to initiate the decelerating rate of responding”) were related to a measure of the reinforcing value of each of the foods. This approach is helpful in the examination of ASR, as it demonstrated that the children who habituated slower also found food more reinforcing than children with a rapid habituation phenotype, thereby providing new insights into possible processes in ASR in childhood.

Boutelle et al. (78) provide a second example of multi-trajectory modeling. They assessed four child (mean age 10.4 years) appetitive traits at 3, 6, 12, and 24 months after baseline. Multivariate GBTM yielded three “trait trajectories of appetitive subgroups.” In a subgroup they labeled “high satiety responsiveness” there was an increasing pattern in satiety responsiveness, a decreasing pattern in food responsiveness and a low stable pattern in emotional eating and negative affect eating. The phenotypes, therefore, were characterized by a different pattern of trajectories for the four eating behaviors. Again, these phenotypes show the potential of multi-trajectory analyses for the investigation of ASR development because they show different combinations of eating behaviors in subgroups of children. The finding that only the high satiety responsiveness subgroup-maintained weight loss following a family-based treatment for children with overweight or obesity provides further support for separating appetite trajectory phenotype subgroups.

### Some Strengths and Limitations of Using Person-Centered Approaches to Understand ASR

The literature includes extensive discussion of assumptions, processes and strategies in model selection, the inclusion of covariates and the limitations of these approaches to person-centered analyses (18, 32, 33, 42, 43, 48, 52, 79, 80). In all FMM, selecting the optimal number of latent subgroups requires the investigation of multiple model fit criteria (62). These may include information criteria statistics [e.g., Bayesian Information Criteria (BIC), Consistent Akaike's Information Criteria (CAIC), and Approximate Weight of Evidence Criterion (AWE)], entropy values, the log-likelihood sample size, and likelihood ratio tests comparing the k-class and k-1 subgroup model [e.g., Bootstrap Likelihood Ration Test (BLRT), Lo-Mendell-Rubin adjusted likelihood ratio test (LMR-LRT)]. Bray and Dziak (52) argue that sometimes the use of FMM/LVMM is something of an art as well as a science in model selection. This is particularly so when the sample size is small relative to the model complexity, or the quality of the measurement model is poor. They suggest that both statistical fit and theoretical interpretability should be considered and at times it is better to select a model with a few interpretable classes even if the statistical fit is not optimal. Herle et al. (32) also comment on “non-science” aspects of model selection. This includes taking account of the size and interpretability of classes (70). Lubke and Luningham (42) in a related way refer to the considerable uncertainty around model selection. Several practical guides to the selection and reporting of latent subgroups are available (81–83). Whilst there is currently there is no “rule-of-thumb” regarding a minimal sample size for FMMs, the impact of sample size on determining the optimal number of latent subgroups in FMM have been investigated in several Monte Carlo simulation studies (81, 84).

In relation to latent transition analysis, there is debate and guidance in the literature about procedures and decisions in the conduct of latent transition analyses including sample size and selection of classes [e.g., (52)]. There are challenges associated with causal analysis using these approaches, as well as the possibility for these analyses to compare different theoretical models and for linking latent classes to predictors and outcomes. Berlin et al. (18) alert researchers to issues in using FMM arising from sample size. They argue that insufficient sample sizes can lead to convergence problems, improper solutions and a limited ability to identify meaningful subgroups. They also point out difficulties in determining adequate sample size, such in relation to reliability and the distribution of variables.

A key advantage of all FMM is the ability to directly incorporate predictors or antecedents, covariates, including time-varying covariates, and outcomes in the model (27, 32, 43, 52, 85, 86). Many techniques have been suggested for the examination of covariates [e.g., (87)]. One possibility is based on a regression model where class membership is predicted by the covariates, or classes are used to predict outcome variables such as zBMI (70). Or, where the covariates are partitioned into the latent classes (32). Here, Herle et al. included a discussion of two approaches to the investigation of covariates and their use. Marsh et al. (88) provided a discussion of the inclusion of correlates: when, why and what it assumes. They argued that their inclusion should not qualitatively change the classes but should make them more accurate and that covariates should be antecedent not concurrent or outcome. The inclusion of covariates in models is complex but a useful discussion is provided by Lubke and Luningham (42) on the theoretical bases of FMM/LVMM and the inclusion of covariates.

Finally, scholarship associated with analytic approaches following a person-centered perspective is an active and expanding field. Authors have discussed additional analysis options or extensions of LCA, LPA, LTA (52), and GMM (60, 62). Bray and Dziak indicate that the FMM/LVMM framework is flexible and permits the specification of different types of mixture models that include path models, factor models, survival models, growth curve models, and that structural equation models can be specified for multiple subgroups.

In summary, and overall, research on ASR-related phenotypes, including trajectory phenotypes (whether using individual constructs or a multitrajectory approach) is contributing to an understanding ASR and its development in childhood. The extensive conceptual and technical literature on person-centered strategies in the investigation of subgroup differences also shows the potential of this approach to contribute to knowledge about ASR and its development in childhood. At the same time, researchers should be cognizant of the assumptions and limitations of this approach, as well as the art vs. science aspects of subgroup identification. An important consideration is also that the phenotypes identified are clearly a product of the number and type of individual variables that have been measured. Further, the inclusion of covariates and outcomes provided opportunities to better understand the predictors or possible origins of ASR, influences on its development and effects on important development outcomes such as BMI and weight gain. Below, we comment further on the importance of phenotype research for advancing knowledge about ASR and its development. We argue for the need to expand measures into new domains of ASR-related constructs.

## Discussion

Knowledge and understanding of ASR can be advanced in many ways: through conceptual and theoretical developments, improvements in research design and methods (especially measurement) and progress in approaches to data analysis and statistics. In this review we have taken a slice through some of these matters via the distinction between person-centered and variable-centered approaches to research, and then a focus on person-centered strategies.

In the discussion we first explore insights from extant person-centered approaches for the conceptualization and measurement of ASR in childhood together with suggestions for future research. Second, we discuss possibilities for combining person-centered and variable-centered approaches in scholarship about ASR and children's eating behaviors in childhood. Third, we argue that person-centered approaches could assist in the design of intervention strategies and in the measurement of intervention outcomes.

### Insights From a Person-Centered Approach for Conceptualizing and Measuring ASR

Research on phenotypes from cross-sectional and longitudinal data has the potential to examine underlying processes and dimensions of ASR in childhood. This is apparent from the labels applied to the latent subgroups [e.g., “high satiety responsiveness” (78), “rapidly decelerating habituation” (77)] and the particular measures used to characterize them in the research reviewed here. This research can also contribute to knowledge about (a) antecedents, precursors, or correlates of ASR-related phenotypes, and (b) associations between phenotypes and outcomes (either developmental outcomes or outcomes from interventions).

Latent class/profile and trajectory analyses have been described as data driven and exploratory [e.g., (43, 89)]. Consistent with this description, findings from person-centered analyses should not be used to infer causality, but rather to generate hypotheses for future testing. Also in line with this description, Bergman and Trost (20) emphasize the vagueness of guiding theories in the case of person-centered approaches, especially about how the studied system operates, for example, about how different measures might interact and coordinate to form distinct phenotypes or how and why there could be different trajectories of ASR and its components. For example, in the Derks et al. (75) study although many patterns were found for each of the measured appetitive traits and these were linked to early life predictors, due to the absence of a clear theoretical foundation for the development of appetitive traits, it is unclear why, for instance, three patterns of emotional eating and 5 patterns for food responsiveness may arise. While recognizing the potential of person-centered approaches for advancing knowledge about ASR and its development, it is important to keep in mind that it needs to be built on sound theory as well as efforts to integrate it with variable-centered approaches. As we mention below, recent data analysis strategies that enable a more confirmatory approach and assessment of predictions are also helpful.

Because person-centered strategies in relation to ASR have been mainly exploratory, an important role of these strategies could be in the development and clarification of conceptualization and measurement. Central to a person-centered approach is the assumption that the individual is an integrated totality over time, with behaviors interwoven and interacting (20). This approach is suited to the exploration of possible developmental mechanisms and to inductive theory building (16). A complexity here is that while ASR could be changing over time, other developmental processes and changes are also occurring. This means that person-centered strategies to investigate the development of ASR will need to take account of wider developmental changes and processes.

Morin et al. (27) suggest that some areas of research might not be sufficiently advanced in theory and with a substantial enough body of results to generate clear hypotheses about the expected nature of profiles. They argue that when this is the case, construct validation is important, through showing that the profiles have heuristic and theoretical values and are meaningfully related to key correlates or outcomes. In this case, confirmatory approaches may be applied. Schmiege et al. (89) discuss approaches to confirmatory latent class analysis, including a dual sample approach and confirmatory testing of a latent class structure. As person-centered evidence on ASR expands, it is possible that these more stringent theoretical tests will become more important.

Fundamentally, phenotypes are suggestive of central components and processes in ASR. But the latent subgroups are limited and constrained by, and entirely reflect, the individual measures used. Person-centered strategies with a focus on ASR-related phenotypes will be better placed to contribute to conceptualization and measurement with the continued addition of individual measures of behaviors, characteristics, traits or processes based on emerging evidence about processes and individual differences in ASR. This includes evidence from biologically based research such as genetic susceptibility, psychoneurological measures, as well as measures about general self-regulation from psychology, neuroscience and from areas such as the effects of highly processed food and the rewarding value of food. When covariates and outcomes are included in the research design, person-centered strategies could progress knowledge about the possible origins of ASR, influences on the development of ASR, and developmental outcome associated with ASR.

Indeed research on ASR-related behaviors, characteristics, traits and processes has expanded considerably in recent years and there is now a growing set of possibilities for inclusion in research about ASR phenotypes in childhood including: temperament (such as impulsivity and effortful control) (90), Executive function (such as inhibitory control), genetic susceptibility, reward sensitivity, hedonic responses to food, cognitive function (91), cognitive control and negative affect (92), state and/or trait food cue reactivity (93), brain reward sensitivity to food cues (94), dietary measures, such as dietary fat or carbohydrates (95, 96), fructose consumption (97), intake of processed food (98), sensory sensitivity (99), neuroimaging functional connectivity (100), metabolomics and analysis of the gut microbiome (101, 102), measures of the social facilitation of eating (103), susceptibility to modeling (104), effects of portion size cues (105) and attachment security (106), behavioral and neural measures of appetitive traits such as through neuroimaging measures (107, 108). A helpful broadening of work on ASR phenotypes is also suggested by attention to endophenotypes where genetic predisposition and neural substrates as well as behavioral measures are included (107, 109–112).

However, the inclusion of many behaviors, characteristics, and traits to determine ASR phenotypes may be too computationally burdensome for the model-based person-centered approaches discussed in the present review. Future ASR research may need to employ machine learning, or data mining, methods to determine phenotypes from large and complex datasets (113–115).

### Combining Person-Centered and Variable-Centered Strategies

Comparisons of person-centered and variable-centered approaches to research have highlighted their differences in assumptions, purpose, sampling, research questions, analytic approach and strengths (16). The two approaches can also be complementary (16, 20, 116). Derks et al. (75) for example, demonstrated how combining the two approaches can provide information about children's ASR. They identified different trajectory patterns of children's eating behaviors using LCGA (person centered) and then investigated the early life or other predictors of those patterns (variable centered). Predictors could include child and family characteristics (51) and BMI-z (50) or socio-demographic and clinical characteristics (117). Another option to combine the two approaches is to first identify latent profiles of ASR and use these as predictors of subsequent outcomes such as BMI or diet [e.g., (118)]. Much of the research on risk factors for obesity or weight gain has examined individual predictor variables. This type of research could be helpfully expanded to include relationships between ASR phenotypes and subsequent measures of weight, thereby gaining greater specificity about potential risks as well as possible processes associated with weight gain and obesity.

Phenotype and other person-centered analyses also enable reflections on theoretical models such as a biopsychosocial approach (119) that incorporate variable and person-centered elements. For instance, these analyses could enable the examination of how biological, psychological and social measures combine in the formation of phenotypes, how they differ from one phenotype to another, and then how the phenotypes relate to model outcomes such as weight gain and adiposity. It would also be possible to incorporate parent and child measures from cross-lagged designs to examine transactional processes via phenotypes and co-variate analyses. Other designs could also provide important insights, such as parallel process latent growth modeling which could investigate ASR trajectories alongside other developmental trajectories, such as BMI or emotion regulation.

In the present review we highlighted the potential of person-centered strategies for the conceptualization and measurement of ASR in childhood and infancy. In contrast to a systematic review, the emphasis of the present review was not on an assessment of the evidence or findings from person-centered strategies. Aligned to the purpose of the review, the literature chosen was supportive rather than critical of these strategies and does not cover nor assess the suitability or quality of all research on person centered approaches to advancing the conceptualization of ASR. We briefly discussed the roles and contributions of person-centered vs. variable centered approaches. There is value in further appraisal of these two approaches to the conceptualization and measurement of ASR in infancy and childhood. Further, as evidence accumulates, there is a need for systematic reviews to appraise and synthesize evidence from the two approaches.

### Person-Centered Strategies in Intervention Design and Measurement of Outcomes

There is scope for person-centered approaches to contribute to the design of intervention strategies and in the measurement of outcomes. Person-centered analyses could better inform intervention strategies by a greater focus on the specificity arising from the identification of phenotype subgroups that could be tied to personalized intervention strategies (44, 78, 117). Phenotype and trajectory analyses could be used to examine intervention outcomes such as through changes in phenotypes following an intervention, and transitions from one phenotype to another, such as has been described in the literature on teaching and learning (40, 120). Finally, in contributing to intervention design and the measurement of outcomes, person-centered approaches could contribute to knowledge about possible developmental processes and assist theory development.

## Conclusion

Person-centered strategies can make an important contribution to advances in the conceptualization and measurement of ASR in children, including to an understanding of developmental paths and processes. This appears to be especially so for person-centered strategies that explore phenotypes, whether based on cross-sectional data or trajectories. The potential contribution seems to be enhanced when combined with variable centered approaches that include predictors or co-variates and that examine outcomes. Possible gains from person-centered approaches should be strengthened by further evidence about individual skills, traits and behaviors that comprise ASR, as well as increased evidence about ASR processes and developmental change/trajectories. There is also a need for overall theory development, more confirmatory research and a greater integration with variable-centered approaches. Finally, evidence about children's appetitive phenotypes and trajectories could assist in the design and measurement of intervention outcomes.

## Author Contributions

The review was initiated by CGR and AR. All authors jointly written, contributed to the article, and approved the submitted version.

## Funding

Funding for open access fees were provided by the School of Exercise and Nutrition Sciences, Deakin University. RL was supported by a National Health & Medical Research Council Emerging Leader Fellowship (APP1175250). The funding bodies had no role in the writing of the manuscript.

## Conflict of Interest

The authors declare that the research was conducted in the absence of any commercial or financial relationships that could be construed as a potential conflict of interest.

## Publisher's Note

All claims expressed in this article are solely those of the authors and do not necessarily represent those of their affiliated organizations, or those of the publisher, the editors and the reviewers. Any product that may be evaluated in this article, or claim that may be made by its manufacturer, is not guaranteed or endorsed by the publisher.

## Abbreviations

FMM: Finite mixture modeling
LCA: Latent class analysis
LCGA: Latent class growth analysis
LPA: Latent profile analysis
LTA: Latent transition analysis
GBTM: Group-based trajectory modeling
GMM: Latent growth mixture modeling
LVMM: Latent variable mixture modeling
CEBQ: Children's Eating Behavior Questionnaire
BMI: Body Mass Index
EAH: Eating in the absence of hunger
ASR: Appetite self-regulation.

## References

[1] Hughes SO Power TG O'Connor TM Orlet Fisher J. Executive functioning, emotion regulation, eating self-regulation, and weight status in low-income preschool children: how do they relate? Appetite. (2015) 89:1–9. 10.1016/j.appet.2015.01.009 25596501 PMC5012640

[2] Saltzman JA Fiese BH Bost KK McBride BA. Development of appetite self-regulation: integrating perspectives from attachment and family systems theory. Child Dev Perspect. (2018) 12:51–7. 10.1111/cdep.12254

[3] Bellisle F Drewnowski A Anderson GH Westerterp-Plantenga M Martin CK. Sweetness, satiation, and satiety. J Nutr. (2012) 142:1149S−54S. 10.3945/jn.111.149583 22573779

[4] Blundell J de Graaf C Hulshof T Jebb S Livingstone B Lluch A. Appetite control: methodological aspects of the evaluation of foods. Obes Rev. (2010) 11:251–70. 10.1111/j.1467-789X.2010.00714.x 20122136 PMC3609405

[5] Forde CG. Measuring satiation and satiety. In: Ares G Varela P editors. Methods in Consumer Research. Cambridge: Woodhead Publishing (2018). p. 151–82.

[6] Gibbons C Hopkins M Beaulieu K Oustric P Blundell JE. Issues in measuring and interpreting human appetite (satiety/satiation) and its contribution to obesity. Curr Obes Rep. (2019) 8:77–87. 10.1007/s13679-019-00340-6 31037612 PMC6517339

[7] Chambers L. Food texture and the satiety cascade. Nutrition Bulletin. (2016) 41:277–82. 10.1111/nbu.1222121452470

[8] Russell A Russell CG. Appetite self-regulation declines across childhood while general self-regulation improves: a narrative review of the origins and development of appetite self-regulation. Appetite. (2021) 162:105178. 10.1016/j.appet.2021.105178 33639246

[9] Wardle J Guthrie CA Sanderson S Rapoport L. Development of the children's eating behaviour questionnaire. J Child Psychol Psychiatry. (2001) 42:963–70. 10.1111/1469-7610.00792 11693591

[10] Bridgett DJ Burt NM Edwards ES Deater-Deckard K. Intergenerational transmission of self-regulation: a multidisciplinary review and integrative conceptual framework. Psychol Bull. (2015) 141:602–54. 10.1037/a0038662 25938878 PMC4422221

[11] Nigg JT. Annual Research Review: on the relations among self-regulation, self-control, executive functioning, effortful control, cognitive control, impulsivity, risk-taking, and inhibition for developmental psychopathology. J Child Psychol Psychiatry. (2017) 58:361–83. 10.1111/jcpp.12675 28035675 PMC5367959

[12] Russell CG Russell A. “Food” and “non-food” self-regulation in childhood: a review and reciprocal analysis. Int J Behav Nutr Phys Act. (2020) 17:33. 10.1186/s12966-020-00928-5 32151265 PMC7063723

[13] Kemps E Goossens L Petersen J Verbeken S Vervoort L Braet C. Evidence for enhancing childhood obesity treatment from a dual-process perspective: a systematic literature review. Clin Psychol Rev. (2020) 77:101840. 10.1016/j.cpr.2020.101840 32172004

[14] Laursen BP Hoff E. Person-centered and variable-centered approaches to longitudinal data. Merrill Palmer Quart. (2006) 52:377–89. 10.1353/mpq.2006.0029

[15] Eye AV Bogat GA. Person-oriented and variable-oriented research: concepts, results, and development. Merrill Palmer Quart. (2006) 52:390–420. 10.1353/mpq.2006.0032

[16] Howard MC Hoffman ME. Variable-centered, person-centered, and person-specific approaches: where theory meets the method. Organ Res Methods. (2018) 21:846–76. 10.1177/1094428117744021

[17] Curran PJ Willoughby MT. Implications of latent trajectory models for the study of developmental psychopathology. Dev Psychopathol. (2003) 15:581–612. 10.1017/S0954579403000300 14582933

[18] Berlin KS Parra GR Williams NA. An introduction to latent variable mixture modeling (part 2): longitudinal latent class growth analysis and growth mixture models. J Pediatr Psychol. (2014) 39:188–203. 10.1093/jpepsy/jst085 24277770

[19] Raufelder D Jagenow D Hoferichter F Drury KM. The person-oriented approach in the field of educational psychology. Problems Psychol 21st Century. (2013) 5:79–88.27247643

[20] Bergman LR Trost K. The person-oriented versus the variable-oriented approach: are they complementary, opposites, or exploring different worlds? Merrill Palmer Quart. (2006) 52:601–32. 10.1353/mpq.2006.0023

[21] Masyn KE. Latent class analysis and finite mixture modeling. In: Little TD editor. The Oxford Handbook of Quantitative Methods in Psychology: Vol 2: Statistical Analysis. 2. Oxford: Oxford University Press (2013). p. 551–611.

[22] Galindo L Power TG Beck AD Fisher JO O'Connor TM Hughes SO. Predicting preschool children's eating in the absence of hunger from maternal pressure to eat: a longitudinal study of low-income, Latina mothers. Appetite. (2018) 120:281–6. 10.1016/j.appet.2017.09.007 28899652 PMC5680110

[23] Gearhardt AN Yokum S Harris JL Epstein LH Lumeng JC. Neural response to fast food commercials in adolescents predicts intake. Am J Clin Nutr. (2020) 111:493–502. 10.1093/ajcn/nqz305 31940031 PMC7049532

[24] Giuliani NR Kelly NR. Delay of gratification predicts eating in the absence of hunger in preschool-aged children. Front Psychol. (2021) 12:650046. 10.3389/fpsyg.2021.650046 33868128 PMC8044964

[25] Miller AL Gearhardt AN Retzloff L Sturza J Kaciroti N Lumeng JC. Early childhood stress and child age predict longitudinal increases in obesogenic eating among low-income children. Acad Pediatr. (2018) 18:685–91. 10.1016/j.acap.2018.01.007 29357310 PMC6067997

[26] Stifter CA Moding KJ. Infant temperament and parent use of food to soothe predict change in weight-for-length across infancy: early risk factors for childhood obesity. Int J Obes. (2018) 42:1631–8. 10.1038/s41366-018-0006-4 29463917 PMC6066452

[27] Morin AJS Bujacz A Gagné M. Person-centered methodologies in the organizational sciences. Organ Res Methods. (2018) 21:803–13. 10.1177/109442811877385633545653

[28] D'Souza NJ Downing K Abbott G Orellana L Lioret S Campbell KJ. A comparison of children's diet and movement behaviour patterns derived from three unsupervised multivariate methods. PLoS ONE. (2021) 16:e0255203. 10.1371/journal.pone.0255203 34314443 PMC8315509

[29] D'Souza NJ Kuswara K Zheng M Leech R Downing KL Lioret S. A systematic review of lifestyle patterns and their association with adiposity in children aged 5-12 years. Obes Rev. (2020) 21:e13029. 10.1111/obr.13029 32297464

[30] DiStefano C Kamphaus RW. Investigating subtypes of child development. Educ Psychol Meas. (2016) 66:778–94. 10.1177/0013164405284033

[31] McLachlan GJ Lee SX Rathnayake SI. Finite mixture models. Ann Rev Stat Appl. (2019) 6:355–78. 10.1146/annurev-statistics-031017-100325

[32] Herle M Micali N Abdulkadir M Loos R Bryant-Waugh R Hubel C. Identifying typical trajectories in longitudinal data: modelling strategies and interpretations. Eur J Epidemiol. (2020) 35:205–22. 10.1007/s10654-020-00615-6 32140937 PMC7154024

[33] Ram N Grimm KJ. Growth mixture modeling: a method for identifying differences in longitudinal change among unobserved groups. Int J Behav Dev. (2009) 33:565–76. 10.1177/0165025409343765 23885133 PMC3718544

[34] Kral TVE Moore RH Chittams J Jones E O'Malley L Fisher JO. Identifying behavioral phenotypes for childhood obesity. Appetite. (2018) 127:87–96. 10.1016/j.appet.2018.04.021 29709528 PMC5994376

[35] Jennings KM Loth KA Tate AD Miner MH Berge JM. Application of latent profile analysis to define subgroups of parenting styles and food parenting practices. Appetite. (2019) 139:8–18. 10.1016/j.appet.2019.04.001 30965046 PMC6556132

[36] Burnett AJ Lacy KE Russell CG Spence AC Worsley A Lamb KE. Groups of mothers based on feeding practices and their associations with dietary quality of pre-school children: a latent profile analysis. Appetite. (2021) 168:105754. 10.1016/j.appet.2021.105754 34666138

[37] Micalizzi L Wang M Saudino KJ. Difficult temperament and negative parenting in early childhood: a genetically informed cross-lagged analysis. Dev Sci. (2017) 20:1–14. 10.1111/desc.12355 26490166 PMC4840089

[38] Blair C Raver CC. Individual development and evolution: experiential canalization of self-regulation. Dev Psychol. (2012) 48:647–57. 10.1037/a0026472 22329384 PMC5264525

[39] Beam CR Turkheimer E. Phenotype-environment correlations in longitudinal twin models. Dev Psychopathol. (2013) 25:7–16. 10.1017/S0954579412000867 23398749 PMC5746192

[40] Hickendorff M Edelsbrunner PA McMullen J Schneider M Trezise K. Informative tools for characterizing individual differences in learning: latent class, latent profile, and latent transition analysis. Learn Individ Differ. (2018) 66:4–15. 10.1016/j.lindif.2017.11.001

[41] Mori M Krumholz HM Allore HG. Using latent class analysis to identify hidden clinical phenotypes. JAMA. (2020) 324:700–1. 10.1001/jama.2020.2278 32808993

[42] Lubke GH Luningham J. Fitting latent variable mixture models. Behav Res Ther. (2017) 98:91–102. 10.1016/j.brat.2017.04.003 28460845 PMC5776694

[43] Nagin DS Odgers CL. Group-based trajectory modeling in clinical research. Annu Rev Clin Psychol. (2010) 6:109–38. 10.1146/annurev.clinpsy.121208.131413 20192788

[44] Acosta A Camilleri M Abu Dayyeh B Calderon G Gonzalez D McRae A. Selection of antiobesity medications based on phenotypes enhances weight loss: a pragmatic trial in an obesity clinic. Obesity. (2021) 29:662–71. 10.1002/oby.23120 33759389 PMC8168710

[45] Hittner JB Faith MS. Typology of emergent eating patterns in early childhood. Eat Behav. (2011) 12:242–8. 10.1016/j.eatbeh.2011.06.001 22051354

[46] Vervoort L Naets T Goossens L Verbeken S Claes L Tanghe A. Subtyping youngsters with obesity: a theory-based cluster analysis. Appetite. (2021) 168:105723. 10.1016/j.appet.2021.105723 34606939

[47] Muthen B Muthen LK. Integrating person-centered and variable-centered analyses: growth mixture modeling with latent trajectory classes. Alcoholism Clin Exp Res. (2000) 24:882–91. 10.1111/j.1530-0277.2000.tb02070.x 10888079

[48] Berlin KS Williams NA Parra GR. An introduction to latent variable mixture modeling (part 1): overview and cross-sectional latent class and latent profile analyses. J Pediatr Psychol. (2014) 39:174–87. 10.1093/jpepsy/jst084 24277769

[49] Leech RM Worsley A Timperio A McNaughton SA. Temporal eating patterns: a latent class analysis approach. Int J Behav Nutr Phys Act. (2017) 14:3. 10.1186/s12966-016-0459-6 28061795 PMC5219683

[50] Boutelle KN Peterson CB Crosby RD Rydell SA Zucker N Harnack L. Overeating phenotypes in overweight and obese children. Appetite. (2014) 76:95–100. 10.1016/j.appet.2014.01.076 24524975 PMC6340290

[51] Tharner A Jansen PW. Kiefte-de Jong JC Moll HA van der Ende J Jaddoe VW. Toward an operative diagnosis of fussy/picky eating: a latent profile approach in a population-based cohort. Int J Behav Nutr Phys Act. (2014) 11:14. 10.1186/1479-5868-11-14 24512388 PMC3922255

[52] Bray BC Dziak JJ. Commentary on latent class, latent profile, and latent transition analysis for characterizing individual differences in learning. Learn Individ Differ. (2018) 66:105–10. 10.1016/j.lindif.2018.06.001

[53] Ryoo JH Wang C Swearer SM Hull M Shi D. Longitudinal model building using latent transition analysis: an example using school bullying data. Front Psychol. (2018) 9:675. 10.3389/fpsyg.2018.00675 29867652 PMC5953336

[54] McMullen J Hickendorff M. Latent variable mixture models in research on learning and individual differences. Learn Individ Differ. (2018) 66:1–3. 10.1016/j.lindif.2018.05.008

[55] Schulz A Leuders T. Learning trajectories towards strategy proficiency in multi-digit division – a latent transition analysis of strategy and error profiles. Learn Individ Differ. (2018) 66:54–69. 10.1016/j.lindif.2018.04.014

[56] Rinne LF Ye A Jordan NC. Development of fraction comparison strategies: a latent transition analysis. Dev Psychol. (2017) 53:713–30. 10.1037/dev0000275 28221051

[57] Muthen L Muthen B. Mplus User's Guide. 8th ed. Los Angeles, CA: Muthen & Muthen (1998-2017).

[58] Pitt E Cameron CM Thornton L Gallegos D Filus A Ng SK. Dietary patterns of Australian children at three and five years of age and their changes over time: a latent class and latent transition analysis. Appetite.s (2018) 129:207–16. 10.1016/j.appet.2018.07.008 30012352

[59] Swanson SA Horton NJ Crosby RD Micali N Sonneville KR Eddy K. A latent class analysis to empirically describe eating disorders through developmental stages. Int J Eat Disord. (2014) 47:762–72. 10.1002/eat.22308 24909947 PMC4211958

[60] Nagin DS. Group-based trajectory modeling: an overview. Ann Nutr Metab. (2014) 65:205–10. 10.1159/000360229 25413659

[61] Hinnant B Schulenberg J Jager J. Multifinality, equifinality, and fanning: developmental concepts and statistical implications. Int J Behav Dev. (2021) 1–14. 10.1177/01650254211020402

[62] Wickrama K Lee TK O'Neal CW Lorenz F. Higher-Order Growth Curves and Mixture Modeling with Mplus. New York, NY: Routledge. (2016).

[63] Gomez R Skilbeck C Thomas M Slatyer M. Growth mixture modeling of depression symptoms following traumatic brain injury. Front Psychol. (2017) 8:1320. 10.3389/fpsyg.2017.01320 28878700 PMC5572290

[64] Becnel JN Williams AL. Using latent class growth modeling to examine longitudinal patterns of body mass index change from adolescence to adulthood. J Acad Nutr Diet. (2019) 119:1875–81. 10.1016/j.jand.2019.04.025 31302035

[65] Lai D Xu H Koller D Foroud T Gao S, A. Multivariate finite mixture latent trajectory model with application to dementia studies. J Appl Stat. (2016) 43:2503–23. 10.1080/02664763.2016.1141181 27642206 PMC5021196

[66] Muthen B. Latent variable analysis: growth mixture modeling and related techniques for longitudinal data. In: Kaplan D editor. The SAGE Handbook of Quantitative Methodology for the Social Sciences. Newbury Park, CA: Sage Publications (2004). p. 345–68.

[67] Hawrilenko M Masyn KE Cerutti J Dunn EC. Individual differences in the stability and change of childhood depression: a growth mixture model with structured residuals. Child Dev. (2021) 92:e343–63. 10.1111/cdev.13502 33423273 PMC8641557

[68] van der Nest G Lima Passos V Candel MJJM van Breukelen GJP. An overview of mixture modelling for latent evolutions in longitudinal data: modelling approaches, fit statistics and software. Adv Life Course Res. (2020) 43:100323. 10.1016/j.alcr.2019.100323 36726256

[69] Den Teuling NGP Pauws SC van den Heuvel ER. A comparison of methods for clustering longitudinal data with slowly changing trends. Commun Stat Simul Comput. (2021) 1–28. 10.1080/03610918.2020.1861464

[70] Herle M Stavola B Hubel C Ferreira DLS Abdulkadir M Yilmaz Z. Eating behavior trajectories in the first 10 years of life and their relationship with BMI. Int J Obes. (2020) 44:1766–75. 10.1038/s41366-020-0581-z 32461555 PMC7610465

[71] Nagin DS. Analyzing developmental trajectories: a semiparametric, group-based approach. Psychol Methods. (1999) 4:139–57. 10.1037/1082-989X.4.2.139 11285809

[72] Norris T Mansukoski L Gilthorpe MS Hamer M Hardy R Howe LD. Early childhood weight gain: latent patterns and body composition outcomes. Paediatr Perinat Epidemiol. (2021) 35:557–68. 10.1111/ppe.12754 33960515

[73] Ventura AK Loken E Birch LL. Developmental trajectories of girls' BMI across childhood and adolescence. Obesity. (2009) 17:2067–74. 10.1038/oby.2009.123 19424165 PMC12908222

[74] van Rossem L Wijga AH Brunekreef B de Jongste JC Kerkhof M Postma DS. Overweight in infancy: which pre- and perinatal factors determine overweight persistence or reduction? A birth cohort followed for 11 years. Ann Nutr Metab. (2014) 65:211–9. 10.1159/000360305 25413660

[75] Derks IPM Bolhuis K Sijbrands EJG Gaillard R Hillegers MHJ Jansen PW. Predictors and patterns of eating behaviors across childhood: results from The Generation R study. Appetite. (2019) 141:104295. 10.1016/j.appet.2019.05.026 31128200

[76] Nagin DS Jones BL Passos VL Tremblay RE. Group-based multi-trajectory modeling. Stat Methods Med Res. (2018) 27:2015–23. 10.1177/0962280216673085 29846144

[77] Epstein LH Carr KA O'Brien A Paluch RA Temple JL. High reinforcing value of food is related to slow habituation to food. Eat Behav. (2020) 38:101414. 10.1016/j.eatbeh.2020.101414 32799072 PMC7484059

[78] Boutelle KN Kang Sim DE Manzano M Rhee KE Crow SJ Strong DR. Role of appetitive phenotype trajectory groups on child body weight during a family-based treatment for children with overweight or obesity. Int J Obes. (2019) 43:2302–8. 10.1038/s41366-019-0463-4 31591483 PMC6858531

[79] Davies CE Glonek GF Giles LC. The impact of covariance misspecification in group-based trajectory models for longitudinal data with non-stationary covariance structure. Stat Methods Med Res. (2017) 26:1982–91. 10.1177/0962280215598806 26282890

[80] Herle M Stavola B Hubel C Abdulkadir M Ferreira DS Loos RJF. A longitudinal study of eating behaviours in childhood and later eating disorder behaviours and diagnoses. Br J Psychiatry. (2020) 216:113–9. 10.1192/bjp.2019.174 31378207 PMC7000294

[81] Nylund KL Asparouhov T Muthén BO. Deciding on the number of classes in latent class analysis and growth mixture modeling: a monte carlo simulation study. Struct Equat Model Multidiscipl J. (2007) 14:535–69. 10.1080/10705510701575396

[82] Weller BE Bowen NK Faubert SJ. Latent class analysis: a guide to best practice. J Black Psychol. (2020) 46:287–311. 10.1177/009579842093093227492449

[83] van de Schoot R Sijbrandij M Winter SD Depaoli S Vermunt JK. The GRoLTS-checklist: guidelines for reporting on latent trajectory studies. Struct Equat Model Multidiscipl J. (2016) 24:451–67. 10.1080/10705511.2016.1247646

[84] Tein JY Coxe S Cham H. Statistical power to detect the correct number of classes in latent profile analysis. Struct Equ Model. (2013) 20:640–57. 10.1080/10705511.2013.824781 24489457 PMC3904803

[85] Twisk J. Is it necessary to classify developmental trajectories over time? A critical note. Ann Nutr Metab. (2014) 65:236–40. 10.1159/000362506 25413664

[86] Francis LA Rollins BY Bryce CI Granger DA. Biobehavioral dysregulation and its association with obesity and severe obesity trajectories from 2 to 15 years of age: a longitudinal study. Obesity. (2020) 28:830–9. 10.1002/oby.22762 32202074

[87] Genge E, A. latent class analysis of the public attitude towards the euro adoption in Poland. Adv Data Anal Classif. (2013) 8:427–42. 10.1007/s11634-013-0156-0

[88] Marsh HW Lüdtke O Trautwein U Morin AJS. Classical latent profile analysis of academic self-concept dimensions: synergy of person- and variable-centered approaches to theoretical models of self-concept. Struct Equat Model Multidiscipl J. (2009) 16:191–225. 10.1080/10705510902751010

[89] Schmiege SJ Masyn KE Bryan AD. Confirmatory latent class analysis. Organ Res Methods. (2018) 21:983–1001. 10.1177/1094428117747689

[90] Saelens BE Melhorn SJ Rowland MG Scholz K De Leon MRB Elfers CT. General and food-specific impulsivity and inhibition related to weight management. Child Obes. (2021) 1–8. 10.1089/chi.2021.0080 PMC8892982 34357785

[91] Smith L Toussaint L Micoli A Lynch B. Obesity, putative biological mediators, and cognitive function in a national sample of children and adolescents. Prev Med. (2021) 150:106659. 10.1016/j.ypmed.2021.106659 34097950

[92] Vainik U Garcia-Garcia I Dagher A. Uncontrolled eating: a unifying heritable trait linked with obesity, overeating, personality and the brain. Eur J Neurosci. (2019) 50:2430–45. 10.1111/ejn.14352 30667547

[93] Reents J Pedersen A. Differences in food craving in individuals with obesity with and without binge eating disorder. Front Psychol. (2021) 12:660880. 10.3389/fpsyg.2021.660880 34149552 PMC8206470

[94] Han P Roitzsch C Horstmann A Possel M Hummel T. Increased brain reward responsivity to food-related odors in obesity. Obesity. (2021) 29:1138–45. 10.1002/oby.23170 33913254

[95] Wallace CW Fordahl SC. Obesity and dietary fat influence dopamine neurotransmission: exploring the convergence of metabolic state, physiological stress, and inflammation on dopaminergic control of food intake. Nutr Res Rev. (2021) 1–16. 10.1017/S0954422421000196 PMC9351269 34184629

[96] Loper H Leinen M Bassoff L Sample J Romero-Ortega M Gustafson KJ. Both high fat and high carbohydrate diets impair vagus nerve signaling of satiety. Sci Rep. (2021) 11:10394. 10.1038/s41598-021-89465-0 34001925 PMC8128917

[97] Payant MA Chee MJ. Neural mechanisms underlying the role of fructose in overfeeding. Neurosci Biobehav Rev. (2021) 128:346–57. 10.1016/j.neubiorev.2021.06.034 34182019

[98] Small DM DiFeliceantonio AG. Processed foods and food reward. Science. (2019) 363:346–7. 10.1126/science.aav0556 30679360

[99] Zickgraf HF Elkins A. Sensory sensitivity mediates the relationship between anxiety and picky eating in children/ adolescents ages 8–17, and in college undergraduates: a replication and age-upward extension. Appetite. (2018) 128:333–9. 10.1016/j.appet.2018.06.023 29928938 PMC8056743

[100] Voigt K Razi A Harding IH Andrews ZB Verdejo-Garcia A. Neural network modelling reveals changes in directional connectivity between cortical and hypothalamic regions with increased BMI. Int J Obes. (2021) 45, 2447–54. 10.1101/2020.05.10.087619 34341471 PMC8528693

[101] Gawlik A Salonen A Jian C Yanover C Antosz A Shmoish M. Personalized approach to childhood obesity: lessons from gut microbiota and omics studies. Narrative review and insights from the 29th European childhood obesity congress. Pediatr Obes. (2021) 16:e12835. 10.1111/ijpo.12835 34296826

[102] Baranowski T Motil KJ Moreno JP. Multi-etiological perspective on child obesity prevention. Curr Nutr Rep. (2019) 8, 1–10. 10.1007/s13668-019-0256-3 30649714 PMC6635107

[103] Ruddock HK Brunstrom JM Higgs PS. The social facilitation of eating: why does the mere presence of others cause an increase in energy intake? Physiol Behav. (2021) 240:113539. 10.1016/j.physbeh.2021.113539 34331957

[104] Blissett J. Effects of modeling on children's eating behavior. In: Lumeng JCFJ editor. Pediatric Food Preferences and Eating Behaviors. London: Academic Press (2018). p. 53–72.

[105] Robertson DA Lavin C Lunn PD. Can visual cues to portion size reduce the number of portions of consumed? Two randomized controlled trials. Ann Behav Med. (2021) 55:746–57. 10.1093/abm/kaaa098 33196083

[106] Beijers R Miragall M van den Berg Y Konttinen H van Strien T. Parent–infant attachment insecurity and emotional eating in adolescence: mediation through emotion suppression and alexithymia. Nutrients. (2021) 13:1662. 10.3390/nu13051662 34068872 PMC8153636

[107] Carnell S Benson L Pryor K Driggin E. Appetitive traits from infancy to adolescence: using behavioral and neural measures to investigate obesity risk. Physiol Behav. (2013) 121:79–88. 10.1016/j.physbeh.2013.02.015 23458627 PMC3725261

[108] Thapaliya G Sadler JR Jansen E Carnell S. Obesity and appetite: evidence for a neurobehavioral model of obesity risk and maintenance. In: Encyclopedia of Behavioral Neuroscience. Second Edition. Elsevier (2021) 347–59. 10.1016/B978-0-12-819641-0.00142-0

[109] Byrne M Tanofsky-Kraff M. Development of loss of control eating. In: Lumeng JCFJ editor. Pediatric Food Preferences and Eating Behaviors. London: Academic Press (2018). p. 233–54.

[110] Goldsmith HH Davidson RJ. Disambiguating the components of emotion regulation. Child Dev. (2004) 75:361–5. 10.1111/j.1467-8624.2004.00678.x 15056191

[111] Kan C Herle M Treasure J Jones A Rijsdijk F Llewellyn C. Common etiological architecture underlying reward responsiveness, externally driven eating behaviors, and BMI in childhood: findings from the Gemini twin cohort. Int J Obes. (2020) 44:2064–74. 10.1038/s41366-020-0605-8 32467612 PMC7610375

[112] Michaud A Vainik U Garcia-Garcia I Dagher A. Overlapping neural endophenotypes in addiction and obesity. Front Endocrinol. (2017) 8:127. 10.3389/fendo.2017.00127 28659866 PMC5469912

[113] Farrahi V Niemela M Karmeniemi M Puhakka S Kangas M Korpelainen R. Correlates of physical activity behavior in adults: a data mining approach. Int J Behav Nutr Phys Act. (2020) 17:94. 10.1186/s12966-020-00996-7 32703217 PMC7376928

[114] Lin Z Feng W Liu Y Ma C Arefan D Zhou D. Machine learning to identify metabolic subtypes of obesity: a multi-center study. Front Endocrinol. (2021) 12:713592. 10.3389/fendo.2021.713592 34335479 PMC8317220

[115] Niemela M Kangas M Farrahi V Kiviniemi A Leinonen AM Ahola R. Intensity and temporal patterns of physical activity and cardiovascular disease risk in midlife. Prev Med. (2019) 124:33–41. 10.1016/j.ypmed.2019.04.023 31051183

[116] Meeusen C Meuleman B Abts K Bergh R. Comparing a variable-centered and a person-centered approach to the structure of prejudice. Soc Psychol Personal Sci. (2017) 9:645–55. 10.1177/1948550617720273

[117] Clairman H Dettmer E Buchholz A Cordeiro K Ibrahim Q Maximova K. Pathways to eating in children and adolescents with obesity. Int J Obes. (2019) 43:1193–201. 10.1038/s41366-018-0271-2 30568266

[118] Cao H Liang Y Li X Zhu L Wu L Liu H. Childhood maltreatment and affective symptoms and severity of drug addiction among Chinese male drug users: variable-centered and person-centered approaches. J Aggress Maltreat Trauma. (2020) 30:1007–27. 10.1080/10926771.2020.1796874

[119] Russell CG Russell A, A. biopsychosocial approach to processes and pathways in the development of overweight and obesity in childhood: insights from developmental theory and research. Obes Rev. (2019) 20:725–49. 10.1111/obr.12838 30768750

[120] Flaig M Simonsmeier BA Mayer A-K Rosman T Gorges J Schneider M. Reprint of “Conceptual change and knowledge integration as learning processes in higher education: a latent transition analysis”. Learn Individ Differ. (2018) 66:92–104. 10.1016/j.lindif.2018.07.001

